# From Biosynthesis to Legislation: A Review of Hydroxytyrosol’s Biological Functions and Safety

**DOI:** 10.3390/ijms26104470

**Published:** 2025-05-08

**Authors:** Zhong Wang, Ziteng Lei, Haijing Zhang, Zheng Liu, Wei Chen, Yan Jia, Ruoyu Shi, Chengtao Wang

**Affiliations:** 1 School of Food and Health, Beijing Technology and Business University, No. 33 Fucheng Road, Haidian District, Beijing 100048, China; wz326wz@163.com (Z.W.); 2431032108@st.btbu.edu.cn (Z.L.); haijingvv@yeah.net (H.Z.); 2330201008@st.btbu.edu.cn (Z.L.); wangchengtao@th.btbu.edu.cn (C.W.); 2 Beijing Advanced Innovation Center for Food Nutrition and Human Health, Beijing Engineering and Technology Research Center of Food Additives, Beijing Technology and Business University, Beijing 100048, China; 3 Beijing Key Laboratory of Plant Resources Research and Development, Beijing Technology and Business University, Beijing 100048, China; 4 Yunnan Plateau Characteristic Agricultural Industry Research Institute, Yunnan Agricultural University, Kunming 650201, China; shiruoy@126.com

**Keywords:** hydroxytyrosol, antioxidant, biosynthesis, safety assessment, regulatory framework, functional foods

## Abstract

Hydroxytyrosol (HT), a potent phenolic compound derived from olives, has attracted significant attention due to its exceptional antioxidant, anti-inflammatory, and antimicrobial properties. This review comprehensively examines recent advances in the synthesis, biological functions, safety profiles, and legal regulations of HT. We discuss both natural and biotechnological synthesis routes, including enzyme-mediated, non-transgenic, and transgenic biosynthetic methods, highlighting recent innovations that have improved yield and purity. The review further explores the multifaceted biological activities of HT, ranging from its role in cardiovascular protection and neuroprotection to its anticancer and metabolic regulatory effects. Safety assessments from animal and human studies are analyzed, demonstrating low toxicity and favorable metabolic profiles at physiologically relevant doses. Additionally, we compare international regulatory frameworks from the United States, China, and the European Union, which underscore the compound’s safe use in food, pharmaceuticals, and cosmetics. Finally, the review outlines future research directions aimed at optimizing production methods, enhancing bioavailability, and addressing long-term toxicological outcomes, thereby reinforcing HT’s potential as a high-value functional ingredient in various industries.

## 1. Introduction

In response to an increasing focus on health and wellness, the food, pharmaceutical, and cosmetic industries are encountering novel challenges. Researchers are persistently exploring natural compounds that offer improved safety and health benefits. HT, a naturally occurring phenolic compound derived from olives, has emerged as a noteworthy candidate due to its significant antioxidant, anti-inflammatory, and antimicrobial properties [1]. Olives, integral to the Mediterranean diet [2], have long been esteemed for their nutritional benefits. In the food sector, HT is utilized as a potent antioxidant that neutralizes free radicals, thereby mitigating the aging process and enhancing overall health [3]. In the medical field, HT has shown potential in addressing cardiovascular diseases, cancer prevention, digestive and metabolic disorders, as well as fibromyalgia [4,5,6,7]. Furthermore, in the field of beauty and cosmetics, Caifeng Chen and colleagues have demonstrated that HT can be used in the treatment of psoriasis [8]. Capitalizing on its antioxidant properties, brands such as Germany’s OLIVEDA and the U.S.-based COSMETIC SKIN SOL have developed skincare products that incorporate HT. Consequently, HT is increasingly integrated into daily consumer products and holds considerable promise for diverse applications.

Recognized as one of the most powerful antioxidants globally, HT is inherently susceptible to oxidation. Its phenolic hydroxyl group, under oxidative conditions, can generate free radicals that initiate further oxidation reactions within the molecule. Factors including exposure to light, elevated temperatures, and oxygen can accelerate this process, leading to the formation of quinones and other oxidation products [9]. These byproducts not only diminish the original antioxidant potency of HT but may also induce adverse side effects. The implications of oxidation are twofold: a reduction in HT’s antioxidant efficacy and a negative impact on the sensory attributes of products due to the formation of dark-colored quinones [10]. In pharmaceutical applications, such degradation compromises therapeutic efficacy. Therefore, implementing effective strategies to protect HT during storage and processing is essential.

## 2. Synthesis Methods of HT

**Figure 1:**
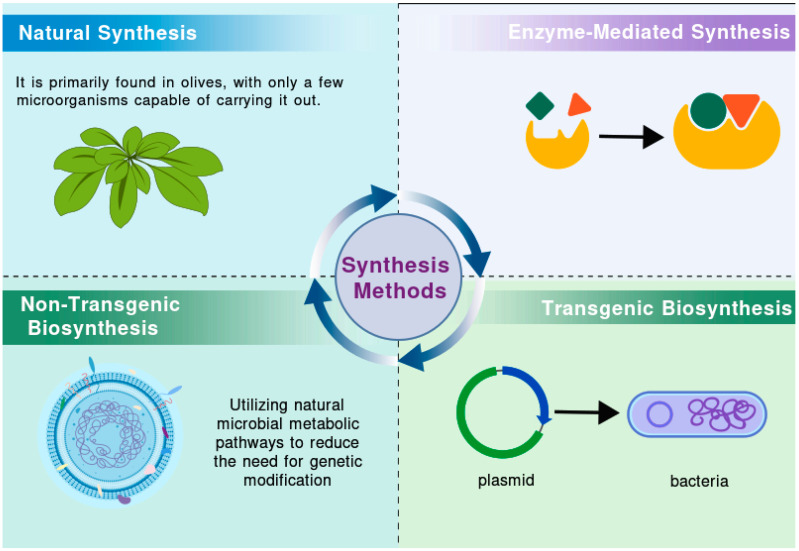
Different synthesis methods of HT.

Given its robust antioxidant properties, HT is widely employed in food, pharmaceutical, and cosmetic formulations. As its applications continue to expand, there is a growing impetus to enhance its production [11]. Olive mill wastewater (OMWW), a major byproduct of the olive oil production process, is generated in large quantities and poses a significant environmental threat. However, it also contains a wealth of bioactive compounds, among which HT has attracted considerable attention due to its notable health benefits [12,13,14]. Various techniques, such as solvent extraction, adsorption methods, membrane separation technologies, and cloud point extraction, can be employed to isolate HT [13,15,16,17]. By optimizing extraction processes and developing new extraction technologies, it is possible to achieve efficient recovery and utilization of HT, providing valuable raw materials for related industries while reducing the environmental impact of OMWW. Nevertheless, OMWW is a complex mixture whose composition varies depending on factors such as olive variety, production methods, climate, and geographical location [18,19]. This variability presents challenges for the extraction of HT. Current technical obstacles include high costs, environmental concerns, the susceptibility of HT to oxidation, and issues related to purity [20,21]. Additionally, consumer acceptance of HT derived from OMWW varies, which could also affect its future market potential. Researchers have thus focused on elucidating the natural synthesis pathways of HT, with the objective of replicating these processes through artificial means. This section reviews various synthesis strategies, including natural synthesis, enzyme-mediated biotransformation, non-transgenic biosynthesis, and transgenic biosynthesis (Figure 1).

### 2.1. Natural Synthesis of HT

Natural synthesis of HT occurs via two primary pathways: plant-based synthesis and microbial fermentation. In olive trees, plant-based synthesis is catalyzed by a series of enzymes—such as polyphenol oxidase (PPO), DOPA decarboxylase (DDC), copper amine oxidase (CuAO), and aldehyde dehydrogenase (ALDH)—as demonstrated by Guodong [22] and Mougiou [23,24]. In these plants, tyrosine serves as the precursor, leading to HT formation through two principal pathways: one that converts L-DOPA and dopamine into HT, and another that utilizes tyramine and 4-hydroxyphenylethanol. Additionally, microbial fermentation has been identified as a viable pathway for HT production. Research on yeast indicates that both the mangiferylic acid pathway and the Ehrlich pathway contribute to HT formation [25]. The process of sugar metabolism, which generates key precursors such as acetyl-CoA, also plays a critical role. Future studies are required to fully delineate the enzyme interactions involved in HT biosynthesis and to optimize microbial fermentation processes to enhance yield. Furthermore, additional research in the food industry is needed to determine optimal methods for maintaining elevated levels of HT in olives.

### 2.2. Enzyme-Mediated Synthesis of HT

Enzyme-catalyzed biotransformation represents a cutting-edge approach to HT production. This method capitalizes on the specificity and efficiency of enzymes to convert structurally related substrates into valuable compounds. As an environmentally sustainable alternative to chemical synthesis, enzyme-based methods can reduce environmental impact while improving product purity (Table 1). Recent studies have focused on enzyme engineering, yielding several viable pathways for HT synthesis, each with distinct advantages and limitations. These advancements not only offer new routes for green production but also broaden the potential for innovative biotechnological applications.

### 2.3. Non-Transgenic Biosynthesis of HT

In contrast to genetically modified approaches, non-transgenic biosynthesis focuses on optimizing conditions in natural microorganisms to enhance HT production. This method leverages innate microbial metabolic pathways, thereby minimizing genetic modifications—a characteristic that aligns well with consumer demands for naturally derived products (Table 2). The non-transgenic approach is not only more environmentally friendly and cost-effective but also sustainable, which has contributed to its popularity in current HT synthesis methodologies.

### 2.4. Transgenic Biosynthesis of HT

Transgenic biosynthesis involves the use of genetic engineering to modify microorganisms for the production of HT (Table 3). This strategy entails the insertion of one or more foreign genes—often from other species—into the genome of the host organism, thereby endowing it with novel capabilities. Various microorganisms, including yeast, *Escherichia coli*, and cyanobacteria, have been utilized in these studies. Ongoing advancements in genetic engineering and process optimization are expected to enhance production efficiency, reduce costs, and ultimately facilitate the commercial production of HT. These innovations hold significant potential for accelerating the application of HT in pharmaceuticals, food, and health supplements.

## 3. Biological Functions of HT

**Figure 2:**
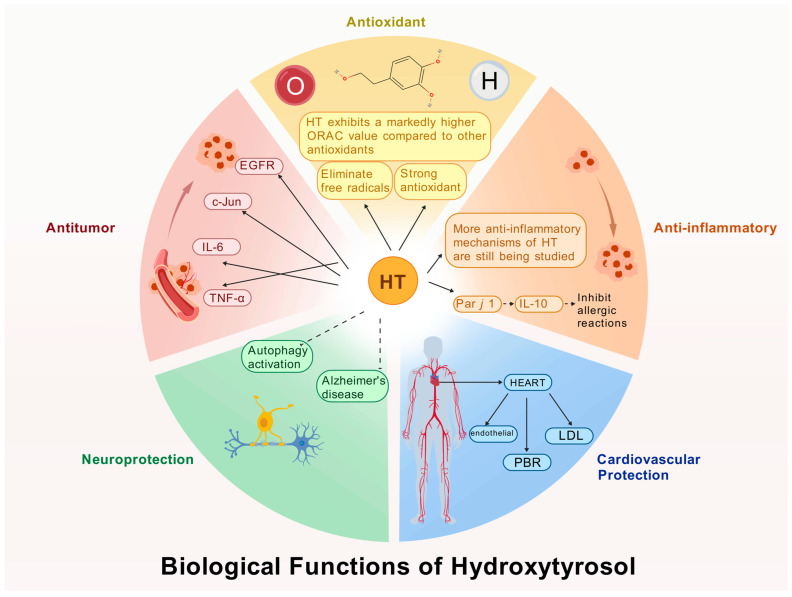
Biological functions of HT.

HT, as a polyphenolic compound, exhibits a range of potent biological activities attributable to its unique molecular configuration. When compared to other natural antioxidants, such as ascorbic acid, glutathione, and vitamin E, HT demonstrates superior antioxidant activity. This section elucidates the multifaceted biological functions of HT, including its antioxidant capacity, anti-inflammatory effects, cardiovascular protection, neuroprotective benefits, and anticancer properties (Figure 2). Collectively, these five domains provide a comprehensive perspective on the biological significance of HT.

### 3.1. Antioxidant Activity

HT is a potent antioxidant capable of protecting cells from oxidative stress. At the cellular and molecular levels, Manna et al. [50] demonstrated that HT can prevent oxidative hemolysis and lipid peroxidation in red blood cells, thereby protecting them from peroxide-induced cytotoxicity. D’Angelo et al. [51] showed that HT effectively prevents UVA-induced skin cell damage through antioxidant activity and protein repair mechanisms. Zhu et al. [52] found that HT enhances antioxidant defenses by regulating the Nrf2 and PPARGC1α pathways in a dose- and time-dependent manner. In animal models, Schaffer et al. [53] confirmed that HT alleviates brain cell toxicity caused by Fe^2+^ and NO in mice, highlighting its protective role against oxidative stress. An increasing number of experimental studies have confirmed HT’s antioxidant capacity, and compared to traditional antioxidants, HT exhibits broader biological activity.

### 3.2. Anti-Inflammatory Activity

HT exhibits significant anti-inflammatory activity across various inflammation models and is considered a safe anti-inflammatory agent [54]. At the cellular and molecular levels, HT effectively inhibits pro-inflammatory cytokines such as tumor necrosis factor-alpha (TNF-α) [55]. It can block the activation of NF-κB—a key transcription factor involved in the regulation of inflammatory gene expression—thereby reducing the transcription of pro-inflammatory mediators and exerting anti-inflammatory effects [56,57]. In in vitro clinical models, Bonura et al. [58] were the first to demonstrate that HT enhances immune tolerance by promoting the secretion of interleukin-10 (IL-10) and modulating allergen-specific immune responses. Numerous similar studies support the conclusion that HT plays a significant role in inflammation regulation and holds potential for the prevention and treatment of various inflammation-related diseases.

### 3.3. Cardiovascular Protection

HT, known for its powerful antioxidant properties, has drawn significant attention for its potential protective role in the prevention and treatment of cardiovascular diseases [59]. In clinical studies, Katogiannis et al. [60] demonstrated that olive extract rich in HT improved endothelial and arterial function by reducing oxidative and inflammatory stress, thereby enhancing left ventricular diastolic function in patients with stable coronary artery disease. Iakovis et al. [61] confirmed that short-term HT supplementation is safe for patients with chronic coronary artery disease and showed trends toward improved diastolic function and aortic elasticity. In a crossover trial, Ikonomidis et al. [62] evaluated the cardiovascular effects of HT in patients with chronic coronary syndrome and observed improvements in endothelial function as well as reductions in oxidative and inflammatory markers. Despite its promising cardiovascular benefits, there are some limitations. For instance, the relatively low bioavailability of HT in the human body may restrict its clinical application [63].

### 3.4. Neuroprotective Effects

HT has shown neuroprotective effects in various neurological diseases and neurophysiological conditions [64]. At the cellular and molecular levels, Leri et al. [65] demonstrated that HT activates autophagy pathways, which helps reduce or prevent neuronal damage associated with neurodegenerative diseases such as Alzheimer’s disease. Moreover, HT significantly promoted the recovery of cell viability after exposure to toxic substances such as Aβ1-42 oligomers. In animal models, Nardiello et al. [66] found that HT supplementation significantly improved cognitive function in mice with Alzheimer’s disease, reduced plaque formation, and decreased inflammation, highlighting its strong neuroprotective potential. Future research should further explore the mechanisms of HT’s neuroprotective action and support the development of more effective HT-based therapeutics, offering hope for patients with neurodegenerative disorders.

### 3.5. Antitumor Activity

HT has demonstrated the ability to inhibit tumor cell growth, induce apoptosis, and regulate the cell cycle in various types of cancer cells [67]. At the cellular and molecular levels, Zhen et al. [68] reported that HT significantly suppresses the growth and proliferation of several tumor cell lines, including breast, gastric, liver, and lung cancer cells. HT affects multiple signaling pathways involved in tumor development and progression. For example, El-Azem et al. [69] found that HT may inhibit estrogen-related pathways (such as EGFR) and regulate the expression of oncogenes such as c-Jun and c-Fos, thereby suppressing tumor proliferation. Notably, Sirangelo et al. [70] were the first to demonstrate HT’s dual function: providing cardioprotection without interfering with the anticancer effects of doxorubicin (Dox), suggesting that HT can protect the heart while maintaining chemotherapeutic efficacy.

As a natural phenolic compound, HT exhibits multiple antitumor mechanisms and has shown promising anticancer activity in both in vitro and in vivo studies. It holds potential as a preventive and therapeutic agent for cancer. Future research should focus on further elucidating HT’s mechanisms of action, optimizing its delivery systems, and evaluating its clinical efficacy through well-designed trials [71].

## 4. Safety of HT

Recognized as one of the most potent antioxidants in nature, HT exhibits a remarkable capacity to neutralize free radicals, outperforming other natural antioxidants such as vitamin E and ascorbic acid. Despite these beneficial properties, the expanding use of HT has prompted critical evaluations of its safety, metabolism, and bioavailability across various application contexts. Current research indicates that HT exhibits low toxicity at physiologically relevant concentrations, as evidenced by both animal models and human trials that report favorable tolerability and minimal adverse effects. Nonetheless, given its increasing application, it is imperative to assess the safety profile of HT under diverse conditions and prolonged exposure scenarios. This chapter examines the digestive, absorptive, and metabolic pathways of HT in both animal models and humans, as well as its toxicity profile, to provide an integrated understanding of its safety for broader applications.

### 4.1. Animal Studies of HT: Absorption and Metabolism

Bai [72] and colleagues conducted a study involving 33 male Wistar rats (distributed into 11 groups, including a blank control) to assess plasma HT levels following oral administration of a 10 mg/mL HT solution (1 mL) under fasting conditions. Plasma samples were collected at predetermined intervals—2, 5, 10, 20, 30, 60, 180, 360, 1440, and 2880 min—and analyzed via GC-MS. The results indicated that HT was detectable in plasma as early as 2 min post-administration, with levels rapidly increasing to a peak between 5 and 10 min, followed by a gradual decline and a sharp decrease after 60 min, rendering HT undetectable by 180 min. These findings underscore the rapid absorption of HT; however, the study did not clarify whether HT is directly excreted or if it exerts its antioxidant effects prior to elimination.

Tuck [73] and collaborators further explored HT metabolism using tritiated HT in male Sprague–Dawley rats. The study compared three administration methods: oral delivery of an HT oil solution, oral delivery of an HT aqueous solution, and intravenous injection of HT in saline. Urine samples collected at 1, 2, 3, 4, 8, and 24 h were analyzed using HPLC with radiodetection. The results revealed that following intravenous injection, HT was excreted within approximately 2 h, while orally administered HT persisted for up to 4 h, with complete elimination observed within 24 h across all methods. Notably, the bioavailability of HT was highest (99%) when delivered in an oil solution, compared to 75% in an aqueous solution. Although this study confirmed systemic absorption and nearly complete bioavailability of HT when administered as an oil solution, the metabolic transformation of HT within the body remained unresolved.

**Figure 3:**
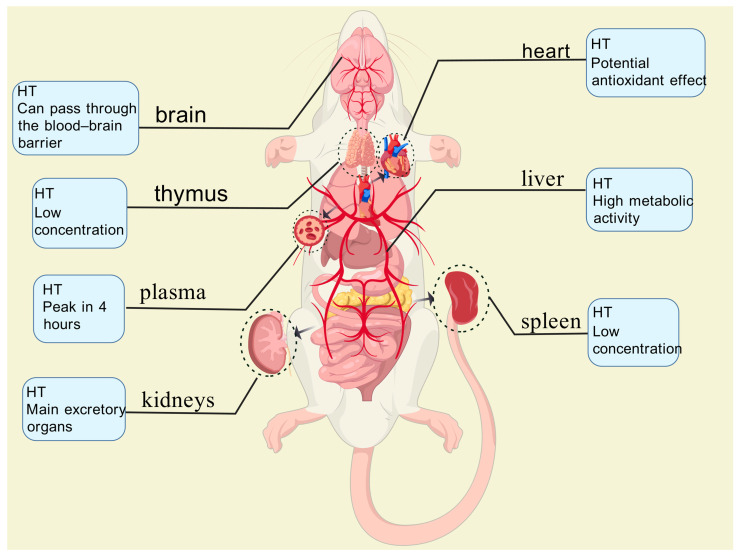
In plasma, oleuropein derivatives reach their peak concentration (24 nmol/L) at 4 h, while phenolic acid metabolites (e.g., homovanillic acid sulfate) remain at lower levels. In the liver, metabolic activity is high, contributing to the conjugation and detoxification of phenolic compounds. The kidneys serve as the primary excretory organs, where metabolite concentrations are highest. In the heart, oleuropein derivatives exhibit potential antioxidant effects, which may help protect arterial walls. In the spleen and thymus, concentrations are relatively low, possibly indicating a role in immune regulation.

Serra et al. [74] administered olive pomace phenolic extracts (PEOC) to Wistar rats via oral gavage at a dose of 3 g/kg body weight. Blood plasma and various tissue samples (heart, brain, liver, kidneys, spleen, testes, and thymus) were collected at 1, 2, and 4 h post-administration and analyzed using ultra-performance liquid chromatography–tandem mass spectrometry (UPLC-MS/MS). Their analysis demonstrated that both the phenolic compounds and their metabolites (primarily HT sulfate esters and glucuronides) were distributed via the bloodstream to multiple tissues (Figure 3), including the brain, indicating their capacity to cross the blood–brain barrier. The liver and kidneys emerged as the primary sites for metabolism and excretion, with sulfation being a predominant metabolic pathway.

### 4.2. Human Studies of HT: Absorption and Metabolism

In human studies investigating HT, it was found that the only trials using pure HT have been conducted by Lopez-Huertas [75]. He studied 14 volunteers with mild hyperlipidemia (baseline total cholesterol 200–239 mg/dL), who were administered 45 mg of pure HT dissolved in 40 mL of saline solution every morning. The aim was to examine the effect of HT on vitamin C levels in the human body. The results showed that HT was well tolerated, with no significant effects on liver function, kidney function, electrolyte balance, or inflammatory markers (such as CRP and VCAM-1). Serum iron levels remained unchanged, but ferritin levels were significantly reduced (*p* < 0.05), suggesting a possible effect on iron storage. Regarding vitamin C, serum levels increased markedly from a baseline of 23.4 μmol/L to 46.4 μmol/L (* *p* < 0.001), with all participants showing increased levels (individual range: 8.4–48.6 → 25.1–72.3 μmol/L). Meanwhile, lipid profiles—including total cholesterol, LDL-C, HDL-C, and triglycerides—showed no significant changes. These findings provide new evidence supporting the potential health benefits of HT through enhancement of the endogenous antioxidant system. However, larger-scale clinical trials are needed to confirm its long-term effects and potential metabolic impact.

In another study, Lopez-Huertas and Gonzalez-Santiago [76] administered HT dissolved in water at a dose of 2.5 mg/kg body weight to 10 volunteers (8 males, 2 females). Plasma and 24 h urine samples were collected and analyzed using gas chromatography–mass spectrometry to identify metabolic products. Plasma lipoproteins (LDL) were also isolated to examine the transient binding of HT to LDL. The study found that HT and its major metabolite, homovanillyl alcohol (HvOH), reached peak plasma concentrations at 13.0 ± 1.5 min and 16.7 ± 2.4 min, respectively. Both levels declined to undetectable values within an hour, indicating rapid elimination. The estimated bioavailability of HT was relatively low (6.2 ± 1.1%) with considerable interindividual variation. Free and conjugated forms of HT and its metabolites were detected in urine, with homovanillic acid (HVA) and 3,4-dihydroxyphenylacetic acid (DHPA) accounting for 31% and 13.2% of total metabolites, respectively. Transient binding between HT and LDL was observed at 10 min, 70% of plasma HT co-precipitated with LDL, and this binding decreased at 20 min, correlating with plasma HT clearance. This study was the first to describe the pharmacokinetics of purified HT in humans, demonstrating its rapid absorption (t_max ≈ 13 min) and low bioavailability (<10%). The transient binding of HT to LDL suggests a potential for localized antioxidant activity; however, no systemic antioxidant effect was observed.

A recent study by Andrea del Saz-Lara [77] has linked HT with exosomes, a topic of growing interest in biomedical research. Exosomes have been increasingly recognized for their potential applications in medicine. In this study, 12 healthy adult volunteers first received a single postprandial dose of HT (25 mg), followed by daily supplementation (25 mg/day) for one week. The goal was to investigate the effects of HT supplementation on plasma extracellular vesicle (EV) secretion, miRNA cargo, and LDL oxidation in healthy individuals. The results showed that HT significantly increased plasma exosome concentration one hour after intake. RNA sequencing analysis revealed that after one week of supplementation, the expression of 55 miRNAs was significantly altered—30 were upregulated, and 25 were downregulated. The upregulated miRNAs were associated with biological processes such as oxygen level response and gland development, while the downregulated miRNAs were linked to processes like ossification and epithelial cell proliferation. Moreover, plasma levels of oxidized LDL (oxLDL) were significantly reduced, further supporting HT’s potential in cardiovascular protection. This study is, to our knowledge, the first to connect the physiological effects of HT with exosomal transport and epigenetic regulation. It reveals a potential mechanism by which HT may exert systemic effects through the exosome–miRNA network. These findings offer a new paradigm for understanding HT’s high efficacy at low doses and open new avenues for the development of exosome-based HT delivery systems and personalized nutritional strategies.

### 4.3. Toxicity and Safety Studies of HT

Auñon-Calles et al. [78] evaluated the toxicity of HT in 80 Wistar Hannover RccHan™ rats (equal numbers of males and females) by administering oral doses of HT at 5 mg/kg/day, 50 mg/kg/day, and 500 mg/kg/day over 13 weeks, with a control group receiving distilled water. Comprehensive evaluations were performed on nearly 40 organs or tissues, including the liver, kidneys, spleen, lungs, heart, and gastrointestinal system. Hematological and biochemical parameters—such as hemoglobin, hematocrit, alanine aminotransferase (ALT), total protein (TP), albumin (ALB), globulin (GLB), blood glucose (GLU), total cholesterol (TC), and triglycerides (TG)—were measured. The study reported no mortality, with only mild salivation observed in the highest dose group, likely attributable to the bitter taste of HT. No significant differences were found in body weight or food intake between the treated and control groups. Although some hematological and biochemical indices exhibited statistically significant differences, these changes were not considered toxicologically significant in the absence of a clear dose–response relationship. Histopathological examinations did not reveal any macroscopic or microscopic tissue alterations, suggesting that HT does not induce genotoxicity or mutagenicity at physiologically relevant concentrations (50 mg/kg bw/day), thereby supporting its use in nutritional supplements and functional foods.

To further assess the genetic toxicity and mutagenicity of HT, Auñon-Calles et al. [79] performed in vitro chromosomal aberration tests using human lymphocytes (with and without metabolic activation via S9 mix) and Ames tests employing various bacterial strains (including Salmonella typhimurium strains TA100, TA98, TA1535, TA1537, and *Escherichia coli* WP2(pKM101)). The chromosomal aberration tests revealed a weak teratogenic effect only at exceedingly high concentrations (10 mM), which are unlikely to be reached under physiological conditions. Similarly, the Ames tests did not detect any mutagenic effects at the concentrations tested, regardless of metabolic activation. Collectively, these findings indicate that HT does not exhibit genetic toxicity or mutagenicity at doses relevant to human consumption.

Overall, existing research suggests that HT exhibits a favorable safety profile, supported by in vitro studies, animal experiments, and human clinical trials. However, under certain specific conditions, potential adverse effects may emerge. For example, a study by Sergio Acín [80] and colleagues used ApoE-deficient mice—a model that naturally develops atherosclerosis even under a low-fat diet—to investigate the effects of HT supplementation in the context of a low-cholesterol diet. The study emphasized the pharmacological actions of HT found in olive oil and raised concerns about its possible detrimental role in atherosclerosis. Contrary to traditional views of HT as an anti-atherosclerotic agent, the findings suggested that under low-cholesterol dietary conditions, HT might actually promote atherosclerosis by altering lipid metabolism and activating monocytes. Similarly, María-Carmen López de las Hazas [81] and co-researchers employed a humanized mouse model to better mimic human lipid metabolism. By administering specific doses of HT, they analyzed its time-dependent effects on lipid profiles and gene expression. Their results indicated that, under certain conditions, HT may induce systemic lipid metabolism disturbances. These effects extended beyond traditional lipid markers and could also involve metabolic regulation through the modulation of miRNA expression. These findings highlight that the biological effects of HT may vary depending on an individual’s metabolic background. As such, caution should be exercised when recommending HT as a dietary supplement. Further clinical and long-term studies are needed to better understand its potential adverse effects. In contrast, a study by M. González-Santiago [82] investigated HT’s effects in atherosclerosis induced by a high-fat, high-cholesterol diet in 64 New Zealand white rabbits. HT supplementation in the experimental group resulted in significant reductions in plasma total cholesterol (TC) and triglycerides (TG), along with a marked increase in HDL cholesterol (HDL-C), suggesting HT’s beneficial potential in improving lipid profiles. Given these conflicting results, the discrepancies likely stem from differences in animal models, dosage and administration methods, study durations, and underlying lipid metabolism mechanisms. Specifically, excessive doses of HT may disrupt lipid homeostasis, while lower doses—or HT delivered within an olive oil matrix—may offer cardiovascular benefits. Therefore, future research should take these variables into account to more accurately evaluate HT’s health effects.

Although long-term exposure to HT and its metabolites cannot be entirely discounted, current data robustly support its safety in the context of nutritional supplements and functional foods. Future research should focus on determining actual exposure levels of HT in the human body during prolonged use, as well as investigating potential interactions with other compounds and its safety across diverse populations.

### 4.4. Metabolism of HT

The metabolism of HT is complex and involves multiple metabolic products, including glucuronide conjugates, sulfate conjugates, homovanillic acid (HVA), and various alcohol derivatives. HT is primarily absorbed through the small intestine [83]. After oral ingestion, it undergoes initial metabolic transformations such as glucuronidation and sulfation [84]. These conjugation reactions occur mainly in the liver and intestinal cells, influencing the bioavailability and biological activity of HT [84,85]. Research has shown that acetylation of HT can enhance its transport across Caco-2 cell monolayers [86].

There are three main metabolic pathways for HT: First of all, conjugation reactions: The primary pathway involves conjugation with glucuronic acid and sulfate to form glucuronide and sulfate conjugates. These reactions increase HT’s water solubility, facilitating its excretion [84,87]. Secondly, methylation: HT can also be methylated to form 3-O-methyl-HT [88]. Finally, oxidative metabolism: HT may undergo oxidative metabolism in the body; however, the specific products and pathways remain to be fully elucidated.

Current research on HT metabolism is largely based on in vitro cell models and animal studies. Further human studies are needed to better understand its metabolic pathways, the bioactivity of its metabolites, and the factors influencing individual variability. This knowledge is essential for accurately evaluating the health benefits of HT and for guiding its application in the food, nutraceutical, and pharmaceutical industries.

## 5. Legal Regulations of HT in Different Countries

**Figure 4:**
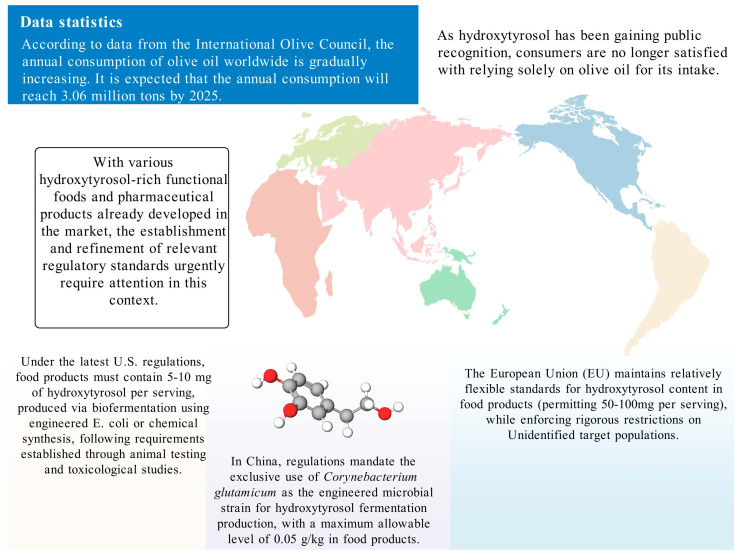
The usage standards of HT in different countries.

HT is generally regarded as safe owing to its origin as a secondary metabolite from olives and its potent antioxidant properties, which make it a suitable additive. This safety profile, together with its high-value potential, has spurred interest in methods to obtain HT at higher purity. However, given the relatively low yield from plant extraction, chemical and biosynthetic methods have been developed to enhance production. Due to the inherent risks associated with these synthesis techniques, many countries have established regulatory frameworks to ensure safe production practices and protect consumer rights (Figure 4).

### 5.1. United States

In 2015, the U.S. Food and Drug Administration (FDA) issued a regulation titled “GRAS-Notice-000600-Hydroxytyrosol” [89], which addresses HT produced via a chemical synthesis route that uses natural dihydroxyphenylacetic acid as a precursor. This process, which involves hydrolysis, esterification, reduction, and purification, yields a product of high purity and yield. The regulation mandates that food servings contain no more than 5 mg of HT. Subsequently, in 2019, the FDA released “GRAS-Notice-GRN-876-Hydroxytyrosol” [90]. This regulation pertains to HT synthesized through fermentation, employing recombinant Escherichia coli BL21(DE3)#145 as a biocatalyst. In this context, the allowable concentration in food products ranges from 5 to 10 mg per serving. The FDA’s approval process was informed by comprehensive animal studies—including subchronic and acute toxicity, genotoxicity—and human research, all of which confirmed the absence of adverse effects at the recommended levels. Additionally, any food products containing HT must comply with existing food regulations.

### 5.2. China

In China, HT is classified as a food additive. In August 2024, the National Health Commission approved HT as a novel food additive, primarily for its antioxidant properties. Furthermore, the Chinese National Center for Food Safety Risk Assessment (CFSA) has established strict standards for HT produced by fermentation using Corynebacterium glutamicum as the catalyst. The final product must exhibit a purity of at least 99%, and its concentration in food products must not exceed 0.05 g/kg. These regulations are notably stringent when compared to those in other regions, partly because CFSA has imposed rigorous limits on microbial counts and heavy metals, such as lead, arsenic, mercury, and cadmium (all below 0.05 mg/kg), to ensure consumer safety.

### 5.3. European Union

According to a scientific opinion issued by the European Food Safety Authority (EFSA) in 2011 [91], HT and its derivatives—such as oleuropein complexes—possess antioxidant properties that help protect low-density lipoprotein (LDL) from oxidative damage. To achieve the claimed health benefit, a daily intake of at least 5 mg of HT and its derivatives (e.g., oleuropein complexes) is required. This amount can be easily obtained through moderate consumption of olive oil. However, the EFSA opinion clearly states that while this health claim is supported by scientific evidence, it does not relate to the prevention or treatment of any specific disease. Rather, it pertains solely to the promotion of general health. As such, any related claims must comply with EU food safety regulations.

The European Union approved HT as a novel food in 2017, confirming its safety for use under defined conditions [92]. Similar to the U.S. regulations established in 2015, HT in the EU is typically produced via chemical synthesis from 3,4-dihydroxyphenylethanol. However, the EU allows for higher concentrations in certain food products; for instance, HT may be added to fish oils and vegetable oils at levels up to 215 mg/kg, and to margarine at up to 175 mg/kg. Notably, EU regulations also impose additional safety precautions, excluding its use by children under 36 months of age, as well as by pregnant and breastfeeding women—reflecting a more conservative regulatory approach.

## 6. Future Research and Outlook

Research on HT, whether derived from natural sources or synthesized via microbial fermentation, has made considerable progress; however, significant challenges remain in translating these findings into scalable industrial applications. HT synthesized through engineered strains of *E. coli* or *C. glutamicum* holds considerable promise for the development of functional foods, pharmaceuticals, and cosmetics. There is an urgent need for efficient, recyclable, and cost-effective production and purification methods to maximize its industrial utility.

Recent advancements in synthetic biology and fermentation technologies have markedly improved HT yields compared to the initial low outputs from plant extraction. Future trends are likely to favor high-yield strains engineered via synthetic biology coupled with cost-effective fermentation techniques. Nevertheless, optimizing the extraction and purification processes remains a challenge. Several promising strategies have been proposed, including membrane technology, liquid–liquid extraction (LLE), and solid-phase extraction using various adsorbents and resins. For instance, Gómez-Cruz et al. [93] achieved an 88.8% recovery rate using ethyl acetate in LLE from olive pomace, yielding 0.6 g/L, while Hadrich et al. [94] reported a 97.53% adsorption rate using modified spherical activated carbon for HT extraction from olive leaves. Further refinement of these techniques is necessary to reduce production costs and improve overall efficiency.

However, issues regarding the stability and bioavailability of HT still need to be addressed. Future research should focus on understanding how HT’s stability varies under different conditions and exploring methods to enhance its stability. Additionally, studying its absorption, distribution, metabolism, and excretion in the human body will help improve its bioavailability. For instance, Zhu et al. [95] demonstrated that HT exhibited superior chemical stability when co-crystallized with nicotinamide or betaine, compared to pure HT. To improve bioavailability, future studies could explore microencapsulation techniques, which would allow HT to be encapsulated in microcapsules, controlling its release in the body and preventing premature release and metabolism in the gastrointestinal tract.

In addition to production challenges, issues related to the stability and bioavailability of HT require further investigation. Future research should focus on characterizing its stability under varying conditions and developing strategies, such as microencapsulation, to enhance its bioavailability by controlling release and protecting it from premature metabolism in the gastrointestinal tract. Moreover, long-term toxicological studies are needed to bridge the current gaps in data, particularly to support its safe use in food and pharmaceutical products. Such studies will be critical in determining chronic exposure levels, potential interactions with other compounds, and ensuring the compound’s safety across diverse populations.

## Figures and Tables

**Table 1:** Enzyme-Mediated Synthesis of HT.

Type	Name	Based on	Acts on	Characteristics	Citation
Non-engineered Enzyme	Yeast glucosidase	Glucosidase produced by Aspergillus niger	Rapeseed leaf extract and olive oil wastewater	Catalyzed the biotransformation of rapeseed leaf extract and olive oil wastewater, successfully extracting HT, with maximum concentrations of 1.1 and 0.5 g/L. By optimizing cultivation conditions, enzyme yield was increased, waste disposal was addressed, and the value of olive oil industry by-products was enhanced.	Hamza and Sayadi [26]
	Immobilized enzyme	Development of a potent biocatalyst by immobilizing β-glucosidase on chitosan-coated magnetic microparticles	Olive leaf extract	This catalyst treated olive leaf extract, achieving over 90% conversion of oleuropein and increasing HT concentration by 2.5 times.	Chatzikonstantinou [27]
	Microwave treatment with enzyme extraction	Combination of microwave treatment and enzyme extraction techniques	Olive	Using pectinase, cellulase, and tannase combined with microwave treatment, the extraction conditions were more favorable for HT release, with a yield of 59.29 mg/kg from pomace.	Macedo [28]
	Thermostable β-glucosidase	Partially purified thermostable β-glucosidase immobilized on a chitosan carrier	Oily leaf extract	This approach enabled the rapid conversion of oily leaf extract into highly purified HT (91–94% by weight) in 14–16 h. The enzyme uses significantly reduced microbial contamination risk and increased reaction speed, thereby improving production efficiency.	Briante [29]
	Marine α-glucosidase	Use of marine α-glucosidase with commercial tyrosinase	Tyrosine glycoside derivatives	Synthesized novel HT monomer and dimer derivatives from tyrosine glycoside derivatives, with final concentrations of 9.35 and 10.8 g/L, respectively.	Trincone [30]
Engineered Enzyme	Protein engineering	Protein engineering and statistical modeling	Toluene monooxygenase	Enhanced substrate specificity and oxidative activity of toluene monooxygenase, enabling efficient synthesis of HT.	Brouk [31]
	Recombinant enzyme application	Biotransformation of non-traditional substrates (e.g., 2-phenylethanol, phthalates, and 2-indanol)	*Escherichia coli*	Recombinant toluene-o-dimethoxybenzene monooxygenase expressed in *Escherichia coli* cells successfully generated six hydroxylated derivatives, including HT.	Donadio [32]

**Table 2:** Non-Transgenic Biosynthesis of HT.

Biological Name	Substrate	Features	Conclusion	Citation
*Serratia marcescens*	p-Coumaric alcohol	Optimized growth conditions and p-coumaric alcohol concentration during the growth of *Serratia marcescens*, significantly increasing HT yield.	*Serratia marcescens* demonstrates high efficiency in converting p-coumaric alcohol to HT, laying a foundation for similar strains to produce HT.	Allouche and Sayadi [33]
*Pseudomonas aeruginosa*	Tyrosol	Used immobilized resting cells of *Pseudomonas aeruginosa* within calcium alginate beads to enhance HT production.	Provides a novel, cost-effective biocatalysis-based method for producing HT, applicable to various microbial systems.	Bouallagui and Sayadi [34]
*Rhodobacter sphaeroides*	Olive mill wastewater	Utilized residual wastewater as raw material, with *Rhodobacter sphaeroides* strain S16-FVPT5 producing a HT-rich mixture.	Addresses the olive mill wastewater issue while transforming waste into the more economically valuable HT.	Carlozzi [35]
Yeast Strain	Tyrosine	Found that the use of yeast strains, combined with an optimized fermentation process, significantly improved HT yield.	Screening identified that commercial yeast strains are the most effective for HT production, revealing the link between microbial metabolism and product yield.	Rebollo Romero [36]
*Rhodococcus pyridinivorans*	Tyrosol or L-Tyrosine	Both wild-type and chemically induced mutant strains utilize tyrosol or L-tyrosine to produce HT, with mutants showing slightly higher yields.	Demonstrates the potential of *R. pyridinivorans* in HT production and highlights the feasibility of non-genetic methods for improving microbial strains.	Anissi [37]

**Table 3:** Transgenic Biosynthesis of HT.

Organism	Substrate	Experimental Process	Reference
*Escherichia coli*	L-Tyrosine	Introduced an artificial pathway into *E. coli* by using mammalian tyrosine hydroxylase (TH) and the endogenous cofactor (MH4) to oxidize L-tyrosine. Endogenous aromatic aldehyde oxidase was also knocked out.	Satoh [38]
	Tyrosine	Developed a hybrid hydroxylase (HpaBC) for production purposes through protein engineering and a directed evolution strategy.	Chen, W. [39]
	Tyrosine	Designed the VanR regulatory protein as a HT biosensor using protein engineering and in vivo optimization. Replaced mammalian tyrosine hydroxylase with *E. coli*’s HpaBC.	Yao, J. [40]
	L-Tyrosine	Engineered a multi-enzyme cascade reaction with HpaBC from *E. coli*, L-amino acid deaminase (LAAD) from *Aspergillus oryzae*, α-ketoacid decarboxylase (ARO10) from *Saccharomyces cerevisiae*, and PAR from *Aspergillus fumigatus*.	Zeng [41]
	Phenol	Integrated the phenol hydroxylase genes (*pheA1* and *pheA2*) from the thermophilic bacterium *Geobacillus thermoglucosidasius* into *E. coli* as a gene fragment.	Orenes-Piñero [42]
	L-Tyrosine	By engineering *Escherichia coli*, two enzyme-coupled pathways, including the dopamine-mediated and keto acid-mediated pathways, were utilized to efficiently synthesize HT (HT) from bio-based L-tyrosine, achieving significant improvements in yield and conversion rate.	Wang, H [43]
*Bacillus licheniformis*	Glucose	Improved phosphoenolpyruvate (PEP) supply by engineering ketoacid decarboxylase, releasing feedback inhibition, and blocking competing pathways.	Zhan, Y. [44]
*Saccharomyces cerevisiae*	Tyrosine or Tyrosol	Improved the ability of *S. cerevisia* to produce HT by heterologously expressing the *E. coli* HpaBC enzyme complex, which hydroxylates tyrosol to HT.	Muñiz-Calvo [45]
	Glucose	Integrated the HpaBC hydroxylase complex from *E. coli* into the genome of *S. cerevisiae*, redirecting metabolism toward tyrosol synthesis.	Bisquert, R. [46]
	Glucose	Overexpressed phenol hydroxylase and employed multi-mode engineering methods to remove tyrosine feedback inhibition by integrating *aro4(K229L)* and *aro7(G141S)* into the genome. Constructed a tyrosine metabolic pathway with AAS enzymes, added *Bbxfpk(op)(t)* to enhance precursor supply, and regulated HT biosynthesis dynamically using the GAL system.	Liu, H. [47]
	L-Tyrosine	Efficient HT biosynthesis from L-tyrosine or simple carbon sources was achieved by converting L-tyrosine to tyrosol via keto acid decarboxylase (e.g., Aro10 from S. cerevisiae) and alcohol dehydrogenase (ADH6), followed by HpaBC-mediated hydroxylation to HT, while feaB knockout minimized 4-HPA accumulation and ADH6 overexpression enhanced tyrosol production.	Liu, Y. [48]
*Saccharomyces cerevisiae*—*Escherichia coli*	Sucrose	Produced tyrosol de novo using the endogenous Ehrlich pathway in *S. cerevisiae*, which was then converted into HT via *E. coli*’s hydroxyphenylacetate 3-monooxygenase (EcHpaBC).	Liu, Y [49]

## Data Availability

Data will be made available on request.
